# Identification of endoplasmic reticulum stress and mitochondrial dysfunction related biomarkers in osteoporosis

**DOI:** 10.1186/s41065-025-00387-7

**Published:** 2025-02-14

**Authors:** Yuxi Chen, Ke Bi, Chunzhi Zhang, Jiaao Gu, Zhange Yu, Jianping Lu, Lei Yu

**Affiliations:** 1 Department of Orthopedic Surgery, The First Affiliated Hospital of Harbin Medical University, Harbin, China; 2 College of Bioinformatics Science and Technology, Harbin Medical University, Harbin, China

**Keywords:** Osteoporosis, Endoplasmic reticulum stress, Mitochondrial dysfunction, Bioinformatics, Machine learning, Molecular docking

## Abstract

**Background:**

Endoplasmic reticulum stress (ERS) and mitochondrial dysfunction (MD) involved in bone metabolism disorders. However, the particular mechanisms of ERS and MD related genes (ERS&MDRGs) in osteoporosis (OP) have not been elucidated. In present study, biomarkers related to ERS and MD in OP were identified.

**Methods:**

Differentially expressed genes (DEGs) were obtained based on GEO dataset. ERS&MDRGs were derived from Genecard database. Initially, ERS&MD related DEGs (ERS&MDRDEGs) were obtained by overlapping DEGs and ERS&MDRGs. The key module was screened by WGCNA. The intersection of ERS&MDRDEGs and key module was screened by machine learning to obtain key genes. Then, receiver operating characteristic curve (ROC) was drawn to calculated diagnostic accuracy of key genes. The ssGSEA and Cibersort algorithms were performed to analyze immune cell infiltration. The miRNA-mRNA-TF network were draw by cytoscape software. Moleculaer docking and DGIdb database were employed for screening potential drugs. Finally, the expression of key genes was verified by qRT-PCR.

**Results:**

The 122 ERS&MDRDEGs were obtained by preliminary screening. ERS&MDRDEGs were mainly enriched in lipid metabolism, calcium ion transport, and ossification. The 5 key genes were identified, including *AAAS*, *ESR1*, *SLC12A2*, *TAF15*, and *VAMP2*. Immune infiltration analysis showed monocyte and macrophage were different between OP and control groups. The miRNA-mRNA-TF regulatory network indicated has-miR-625-5p, has-miR-296-3p, CTCT and EP300 as potential regulatory targets. The 2 potential small molecule drugs, namely bumetanide and elacestrant were screened. The expression of *AAAS*, *ESR1*, *VAMP2* were higher, and *SLC12A2* and *TAF15* were lower in OP than control group.

**Conclusion:**

This research identified 5 key genes *AAAS*, *ESR1*, *SLC12A2*, *TAF15* and *VAMP2*. Bumetanide and elacestrant were potential drugs. These findings provided valuable insights into the pathophysiology of OP and the development of new therapeutic strategies.

**Supplementary Information:**

The online version contains supplementary material available at 10.1186/s41065-025-00387-7.

## Introduction

Osteoporosis (OP) is a metabolic bone disease marked by lower bone mineral density (BMD) and bone microstructure deterioration, leading to reduced bone strength and elevated fracture risk [1, 2]. OP occurs mostly in postmenopausal women and older men [3]. Both inherited and environmental factors participate in the occurrence of OP [4]. Accumulation of unfolded/misfolded proteins in endoplasmic reticulum can activate endoplasmic reticulum stress (ERS) [5]. Factors such as metabolic disorders, aging and adverse drug reactions triggers of ERS [6]. Studies have reported that ERS regulates osteoblast viability, osteogenic differentiation and osteoclast function [7]. Currently, developing therapeutic agents targeting against ERS to inhibit OP has received the attention from numerous scholars [7]. Meanwhile, healthy mitochondria are essential for maintaining the balance between osteogenesis and osteoclasis, and mitochondrial dysfunction (MD) disrupts this balance and promotes OP progression [8]. MD is exacerbated by the mitochondrial electron transport chain uncoupling, decreased ATP synthesis, increased reactive oxygen species, and abnormal mitochondria accumulation [9]. Existing studies have confirmed that ameliorating OP by modulating mitochondria has promising application [10]. Endoplasmic reticulum and mitochondria can complete direct signaling through direct binding, as well as indirect signaling through reactive oxygen species, signaling pathways and inflammatory cytokines, forming a feedback loop [11, 12]. However, there are few studies focusing on both ERS and MD regarding OP. The aim of this study is to screen the key genes, potential drugs for ERS and MD in OP.

Early bone loss is asymptomatic and difficult to detect before fracture. The clinical diagnosis of OP relies on dualenergy X-ray absorptiometry (DXA) which is insensitive to early bone loss [13]. OP is mainly treated with drugs, including bisphosphonates, teriparatide and romosuzumab, but all have limitations and adverse effects [14]. New drug development is risky, expensive, and time-consuming. High-throughput screening and bioinformatics analysis have been used in recent years to screen key genes and biomarkers with promising results [15]. Molecular docking can evaluate the possibility and stability of small molecule drug binding to proteins [16]. Therefore, this study used bioinformatics analysis, machine learning to screen biomarkers related to ERS and MD in OP, and molecular docking to screen potential drugs, providing a basis for biomarker identification and drug discovery.

In this study, the expression profile of OP was obtained from GEO database, and endoplasmic reticulum stress related genes (ERSRGs) and mitochondrial dysfunction related (MDRGs) were obtained from Genecard database. ERS&MD related DEGs (ERS&MDRDEGs) were enriched and analyzed. Key module genes were screened by WGCNA. Then, key genes were screened by machine learning, and ROC curves were drawn to evaluate diagnostic efficiency of key genes. The network of miRNA-mRNA-TF was builted to understand regulatory mechanisms of key genes. Small molecule drugs from DGIdb database were further screened by molecular docking. Finally, the expression of key genes was verified by bone loss model mice. This study provides new biomarkers for early diagnosis, new potential drugs, and further understanding of the pathophysiology of OP.

## Materials and methods

### Data acquisition

The GSE35959, GSE7158, GSE56814 and GSE56815 datasets were downloaded from Gene Expression Omnibus database (GEO, https://www.ncbi.nlm.nih.gov/geo/) (Table. S1). This study focused on human bone marrow mesenchymal stem cells, and GSE35959 was selected as the training set due to its largest sample size. Meanwhile, GSE7158, GSE56814 and GSE56815 serve as validation dataset. ERSRGs and MDRGs derived from GeneCards (https://www.genecards.org/) with correlation score > 3.

### Identification of ERS&MDRDEGs

DEGs between OP and control were identified by “limma” package with|log FC| >1 and *P*. adjust < 0.05 [17]. ERS&MDRDEGs were obtained by Venn diagram of ERS&MDRGs and DEGs.

### GO and KEGG pathway enrichment analysis

The “clusterProfiler” R package was applied to annotate gene ontology functions of ERS&MDRDEGs, and to screen for potentially signaling pathways [18]. Some interested gene ontology functions and signaling pathways with *P*. adjust < 0.05 were visualized.

### Weighted gene co-expression network analysis

The WGCNA was utilized to identify highly synergistically varying gene sets and to identify candidate biomarkers or therapeutic targets based on correlation between gene sets and phenotype [19]. The ERS&MD scores were calculated by “GSVA” package as sample trait [20]. The “WGCNA” package was utilized to perform weighted gene co-expression network analysis (WGCNA) [21]. Module-trait correlations were assessed by pearson correlation analysis. Modules were defined as key modules that were highly correlated with both ERS&MD and OP.

### Correlation analysis of candidate genes

The correlation analysis of 16 candidate genes was performed by “corrplot” package. Correlation coefficients among candidate genes were calculated, with blue representing positive correlation and red representing negative correlation.

### Protein-protein interaction network

Protein-protein interaction (PPI) networks of 16 candidate genes was constructed by STRING database (https://cn.string-db.org/, version 12.0) [22]. Meanwhile, the MCC algorithm in the cytoHubba plugin (Cytoscape version 3.10.1) served to screen hub genes and visualize them [23].

### Friend analysis

Friend analysis is a network topology-based analysis approach for exploring genes interrelationship and roles in biological processes. The internal correlations of 16 candidate genes was analyzed by “GOSemSim” package [24].

### Machine learning

The least absolute shrinkage and selection operator (LASSO) regression analysis could reduce model complexity, prevent model overfitting, and improve generalization while ensuring the best fit error [25]. The LASSO regression analysis was carried out on 16 candidate genes by “glmnet” package, and 7 key genes were derived after 10-fold cross-validation [26]. Feature variable screening in support vector machine-recursive feature elimination (SVM-RFE) could screen the best variables also known as feature genes based on feature vectors [27]. The SVM-RFE was done by “e1071” package [26]. When the generalization error was minimum, the number of features was 8. The RandomForest (RF) was often used for model construction by counting the predictions of each decision tree in the forest and selecting the final result from these predictions based on a voting method [28]. The predictive accuracy and Gini coefficients of the ERS&MDRDEG were calculated by “randomForest” package and the top10 were ranked [29].

### ROC curve

The receiver operating characteristic curve (ROC) of key genes were drawn by “pROC” package (Version 1.18.5). The area under the curve (AUC) was calculated to evaluate diagnostic efficiency of key genes.

### GSEA of key genes

The correlation coefficients between key genes and remaining genes were calculated. Based on the list of remaining genes, gene set enrichment analysis (GSEA) of key genes were performed by “clusterProfiler” package [18]. The top 5 entries were visualized.

### Immune infiltration analysis

The Cibersort and single-sample GSEA (ssGSEA) algorithms were utilized to assess the immune infiltration [30, 31]. The different immune cell abundances was assessed by “GSVA” package [20]. The percentage and abundance of immune cells were calculated by “Cibersort” package. Correlations between key genes and immune cells were calculated by the “spearman” algorithm.

### The mRNA- miRNAs, TFs-mRNA and mRNA-Drugs network

The starBase (https://rnasysu.com/encori/) and miRWalk databases (http://mirwalk.umm.uni-heidelberg.de/) were employed to forecast miRNAs of key genes. The CHIPBase (https://rnasysu.com/chipbase3/index.php) and hTFTarget database (https://guolab.wchscu.cn/hTFtarget/) were utilized to predict TFs for key genes. The DGIdb database (https://www.dgidb.org/, version 5.0.6) predicted potential drugs for OP (criteria: interaction score > 0.5 and FDA approval). The cytoscape soft was taken to visualize the above results.

### Molecular docking

Molecular docking was used to study molecular affinity between small molecules and proteins. The protein crystal structures were downloaded from PDB database, and 3D structures of small molecules were downloaded from the PUBCHEM database. We performed molecular docking work by employing AutoDock Vina 1.1.2 software [32]. Prior to docking, PyMol 2.5 was used to process all receptor proteins. Compare the magnitude of binding energies, analyze the sites where small molecules bind to proteins, the number of binding hydrogen bonds and thus assess the binding stability between small molecules and proteins.

### Bone loss model mice

The ovariectomy (OVX) is a well-established animal model for simulating estrogen deficiency-induced OP. The 8-weeks female C57BL/6J mice were procured from Liaoning Changsheng Biotechnology Co. and randomly divided equally into two groups. After 1 week of acclimatization, the mice underwent OVX or sham operation. The procedure is briefly described as follows: anesthesia, depilation, disinfection, tissue separation, ovary exposure and removal, wound closure. All mice were kept under identical pathogen-free conditions with adequate food and water. After 8 weeks of operation, the mice were euthanized and femurs were obtained for subsequent studies.

### Quantitative real-time PCR (qRT-PCR)

Total RNA of bone tissue was extracted using TRIzol reagent (Invitrogen, California, USA). 500 ng total RNA were then reverse transcribed to cDNA in a total reaction volume of 10 µL by cDNA Reverse Transcription Kit (Thermo Fisher Scientific, Waltham, Massachusetts, United States). SYBR Green PCR Master Mix (Applied Biosystems, USA) was used for real-time quantitative PCR. The primer sequences were as follows:

**Table:** 

Genes	Forward Primer (5’-3’)	Reverse Primer (5’-3’)
ESR1	TCTGCCAAGGAGACTCGCTACT	GGTGCATTGGTTTGTAGCTGGAC
AAAS	GGCAGCAAAGTCCTGGCTACTA	AGCAGTCGGTTTCCATCTGGAC
SLC12A2	GATTCGCAGAGACTGTGGTGGA	CTCCATTCCAGCCACTGAGATG
TAF15	TGCAATGAGCCTAGACCAGAGG	CACTCCTGTCTCCACCATAACC
VAMP2	GAGCTGGATGACCGTGCAGATG	ATGGCGCAGATCACTCCCAAGA
18s	GGCGGCTTGGTGACTCTAGATAAC	CCTGCTGCCTTCCTTGGATGTG

### Statistical analysis

The experiment data were displayed by mean ± SD and replicated at least three times. All statistical analyses were done in R software (version 4.3.3). The *P* < 0.05 was considered statistically significant.

## Results

**Fig. 1:**
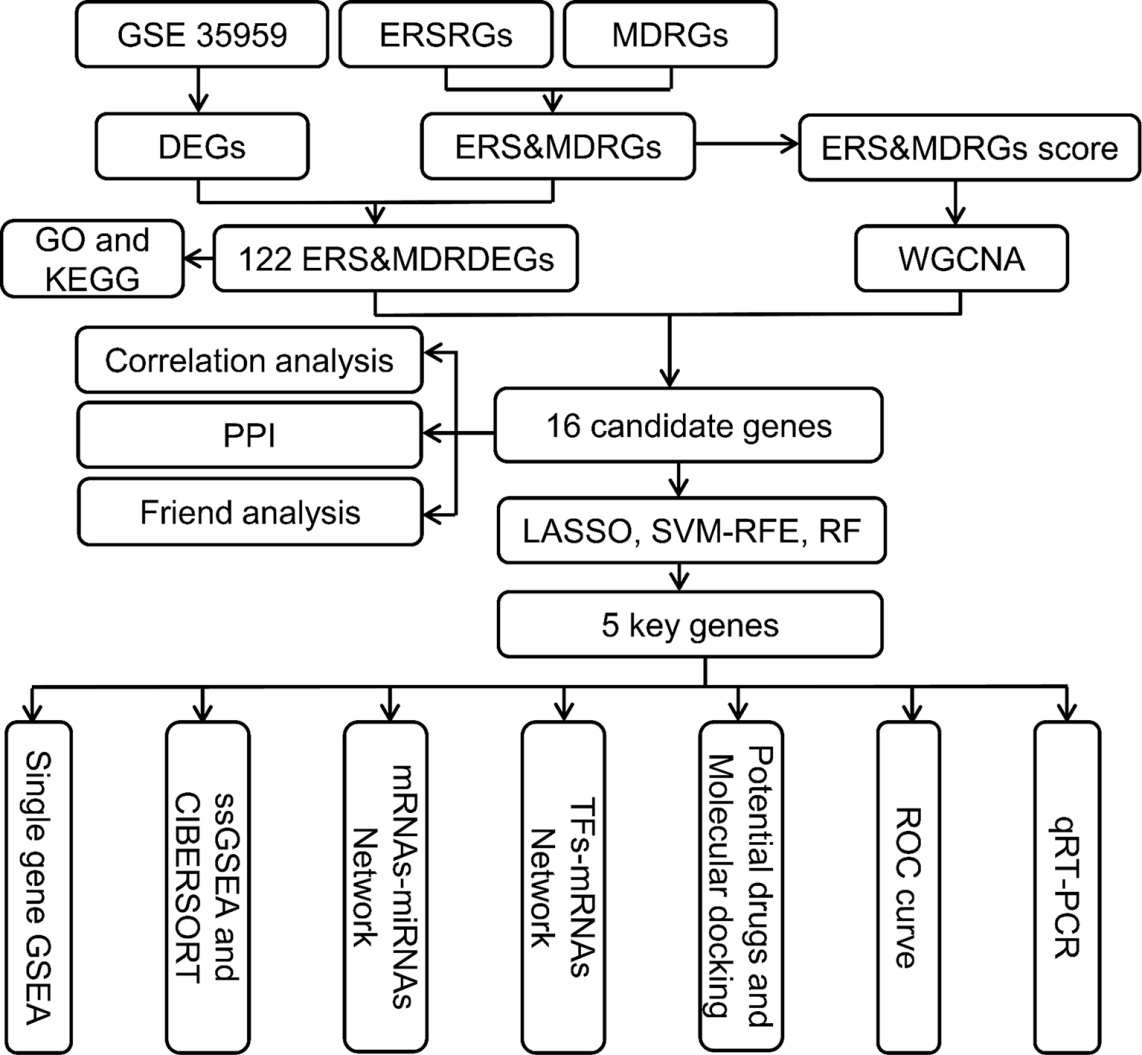
The flow chart of this research

The flowchart of this research was presented in Fig. 1.

### Identification of DEGs

The GSE35959 dataset was normalized for correction, and PCA analysis was performed for subsequent analysis (Fig. S1A-B, S1C-D). DEGs between OP and control were screened with|log FC|>1 and *P*. adjust < 0.05. DEGs included 309 highly expressed genes and 435 lowly expressed genes and were visualized by volcano plot and heatmap (Fig. S1E and S1F).

### Identification of ERS&MDRDEGs and function enrichment

**Fig. 2:**
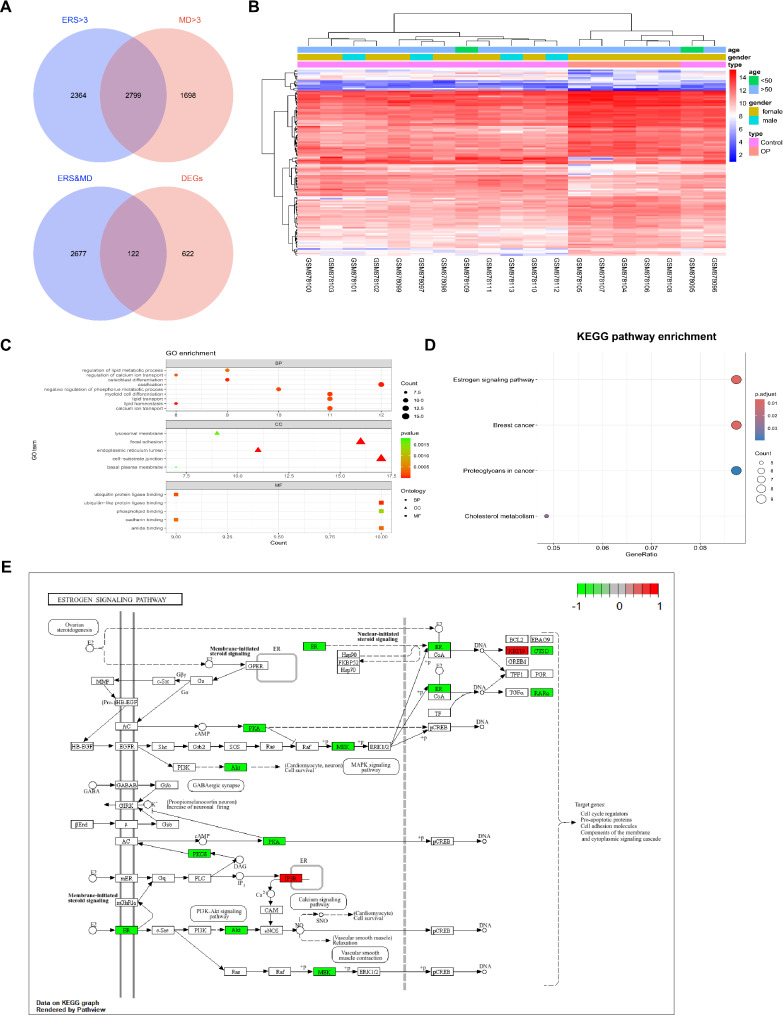
The identification and enrichment analysis of ERS&MDRDEGs. (**A**) The Venn plots of ERSRGs and MDRGs and DEGs. The Venn plots of ERS&MDRGs and DEGs. (**B**) The Heatmap of ERS&MDRDEGs. (**C**) The GO enrichment of ERS&MDRDEGs. (**D**) The KEGG pathway enrichment of ERS&MDRDEGs. (**E**) The ERS&MDRDEGs regulation of estrogen signaling pathways. Red represents upregulation and green represents downregulation. Endoplasmic reticulum stress and mitochondrial dysfunction related differentially expressed genes, ERS&MDRDEGs

ERSRGs and MDRGs were gained from GeneCards database with relevance score > 3. The 2799 ERS&MDRGs were obtained from venn diagram of ERSRGs and MDRGs (Table. S2). The 122 ERS&MDRDEGs were acquired from venn diagram of DEGs and ERS&MDRGs (Fig. 2A). The heatmap showed the expression of ERS&MDRDEGs in OP and control group (Fig. 2B). GO and KEEGG enrichment analyses were done for ERS&MDRDEGs. For GO terms, biological processes (BP) were enriched for ossification, calcium ion transport, lipid metabolism, osteoblast differentiation. The cellular components (CC) were enriched for cell-substrate junction, focal adhesion and endoplasmic reticulum lumen. The molecular functions (MF) were enriched for ubiquitin-like protein ligase binding, phospholipid binding and amide binding (Fig. 2C). For KEGG pathway, ERS&MDRDEGs enriched in estrogen signaling pathway, breast cancer, proteoglycans in cancer and cholesterol metabolism (Fig. 2D). The ERS&MDRDEGs regulation of estrogen signaling pathway was demonstrated (Fig. 2E). In general, ERS&MDRDEGs were enriched in crucial biological processes and pathways related to osteogenesis, such as osteoblast differentiation, lipid metabolism, calcium ion metabolism and estrogen signaling pathways.

### Weighted gene co-expression network analysis

**Fig. 3:**
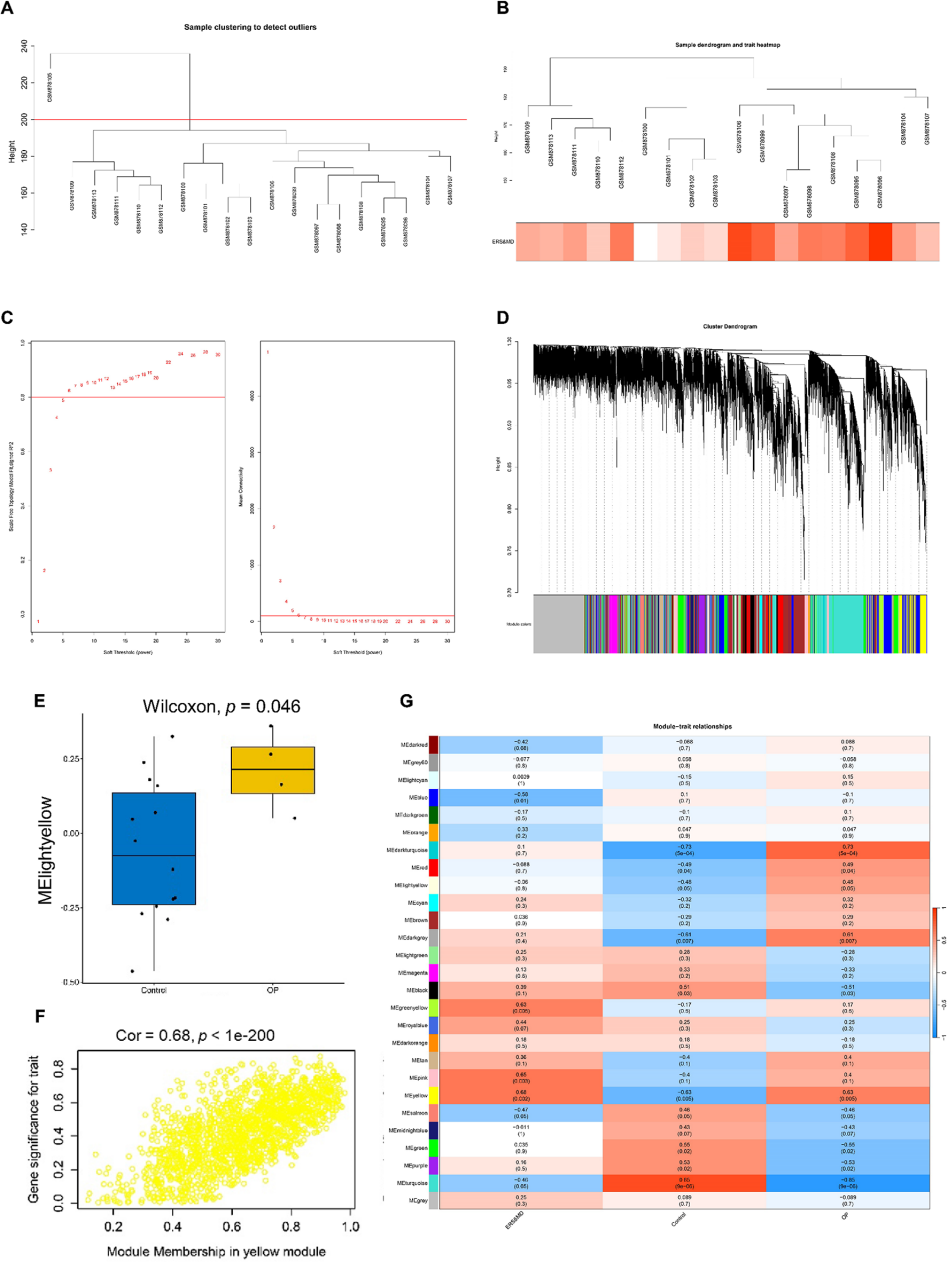
Weighted gene co-expression network analysis. (**A**) Detection of outlier samples by sample clustering. (**B**) Sample dendrograms and trait (ERS&MD) heatmap. (**C**) Select the best soft threshold in WGCNA. The left figure shows the optimal soft threshold and the right figure shows the network connectivity in different soft threshold cases. (**D**) Clustering of gene modules. The Upper part shows the cluster dendrogram and the lower part shows the gene module. (**E**) Boxplot plot of yellow module eigengene. (**F**) The Scatterplot of correlation between yellow module genes and ERS&MD. (**G**) Heatmap of correlations between module genes and ERS&MD or OP

The ERS&MD scores were calculated by “GSVA” package and served as sample trait for subsequent WGCNA (Table. S3). The samples were clustered with a threshold value of 200 to exclude outlier samples (Fig. 3A). The ERS&MD scores served as sample traits and the samples were re-clustered (Fig. 3B). When selecting the optimal soft threshold, the network was constructed (Fig. 3C). When the minimum module gene number was set to 50, 27 modules were obtained by hierarchical clustering after merging (Fig. 3D). The yellow module eigengene was markedly different between OP and control group (Fig. 3E). Meanwhile, yellow module genes showed a strong positive correlation with trait (Fig. 3F). The correlation heatmap showed that yellow module has a correlation coefficient 0.68 with ERS&MD (*P* = 0.002) and 0.63 with OP (*P* = 0.005) (Fig. 3G). The yellow module contained 1536 genes (Table. S4) and served as the key module.

### Candidate genes correlation analysis and PPI network

**Fig. 4:**
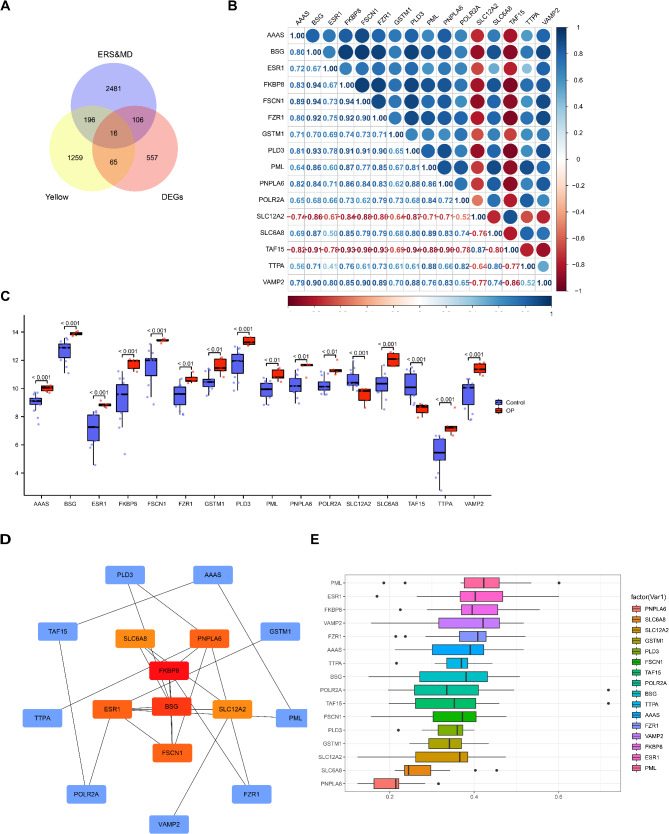
The correlation analysis and PPI networks of candidate genes. (**A**) The Venn plots of ERS&MDRGs, yellow module and DEGs. (**B**) The correlation heatmap of 16 candidate genes. Red represents positive correlation and blue represents negative correlation. (**C**) The grouping comparison chart of 16 candidate genes expression. Blue represents the control and red represents OP. (**D**) PPI networks of 16 candidate genes. The nodes represent proteins and the connecting lines represent interactions between proteins. (**E**) Friend analysis of 16 candidate genes. Vertical markers represent candidate genes and horizontal markers represent correlation coefficients

The 16 candidate genes were remained by venn diagram of ESR&MDRDEGs and yellow module genes (Fig. 4A). Most of correlation coefficients in absolute value among candidate genes were > 0.6 (Fig. 4B). All candidate genes were significantly differentially expressed between OP and control (Fig. 4C). The PPI networks of candidate genes was constructed to analyze potential interactions of candidate genes at protein level. The hub genes were screened by MCC algorithm, including *FKBP8*, *BSG*, *FSCN1*, *PNPLA6*, *ESR1*, *SLC12A2* and *SLC6A8* (Fig. 4D). Friend analysis showed that *PML*, *ESR1*, *FKBP8*, *VAMP2*, *FZR1*, *AAAS* and *TTPA* correlated strongly with other candidate genes (Fig. 4E).

### Key genes screening and ROC curve

**Fig. 5:**
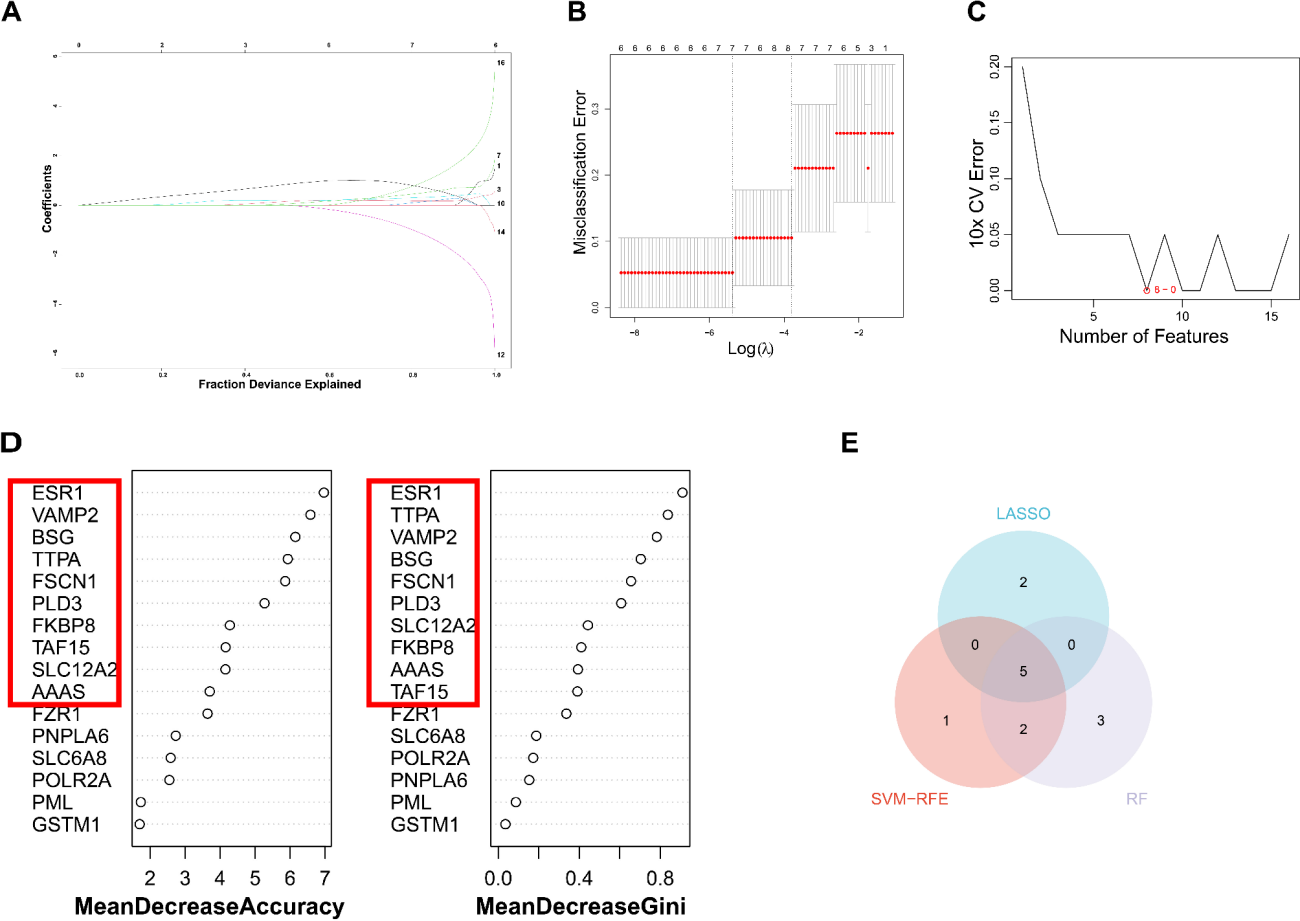
Identifying key genes through machine learning. (**A**) The Coefficient trace graph in LASSO regression analysis. (**B**) LASSO logic coefficient penalty diagram. (**C**) Prediction accuracy and number of features relationship in SMV-RFE. (**D**) MeanDecreaseAccuracy and MeanDecreaseGini scatterplot of candidate genes in RF. (**E**) The venn plot of LASSO, SVM-RFE and RF. The least absolute shrinkage and selection operator, LASSO; Feature variable screening in support vector machine-recursive feature elimination, SVM-RFE; RandomForest, RF

LASSO, SVM-RFE and RF were utilized to screen key genes. The 7 key genes were available after selecting best lambda values in LASSO (Fig. 5A and B). When minimizing the generalization error in SVM-RFE, the number of features was 8 (Fig. 5C). In RF, candidate genes were ranked according to prediction accuracy and Gini coefficient, and top 10 genes were picked out (Fig. 5D). The venn diagram of 3 machine learning showed that key genes included *AAAS*, *ESR1*, *SLC12A2*, *TAF15* and *VAMP2* (Fig. 5E and Table. S5). The ROC curve of key genes showed that key genes had favorable diagnostic performance in training and validation datasets (Fig. S2).

### GSEA of key genes

The 5 key genes were analyzed by single genes GSEA. GO analysis showed that *AAAS* expression was positively correlated with organelle localization, chromosome segregation and DNA replication regulation (Fig. S3A). *ESR1* expression showed negative association with actin and positive association with cellular translation and ribosomes (Fig. S3B). *SLC12A2* expression related positively to chromosome segregation and chromatin regions (Fig. S3C). *TAF15* was positively associated with chromosome alignment, mitosis and chromosome segregation (Fig. S3D). *VAMP2* was negatively related to cellular translation and ribosomes (Fig. S3E).

KEGG pathway enrichment showed that *AAAS* was negatively correlated with translation initiation, ribosomes and translation elongation (Fig. S3A). *ERS1* showed positive association with translation initiation, ribosomes and translation elongation (Fig. S3B). *SLC12A2* showed negative correlation with translation initiation, ribosomes and translation elongation (Fig. S3C). *TAF15* positively correlated with mitosis, chromosome degradation and chromosome segregation (Fig. S3D). *VAMP2* was associated negatively with translation initiation, translation elongation and ribosomes (Fig. S3E). Overall, these key genes major molecular functions included mitosis, chromosome segregation and cellular translation, and were engaged in pathways such as eukaryotic translation initiation, translation elongation and ribosome regulation.

### Immune infiltration analysis

**Fig. 6:**
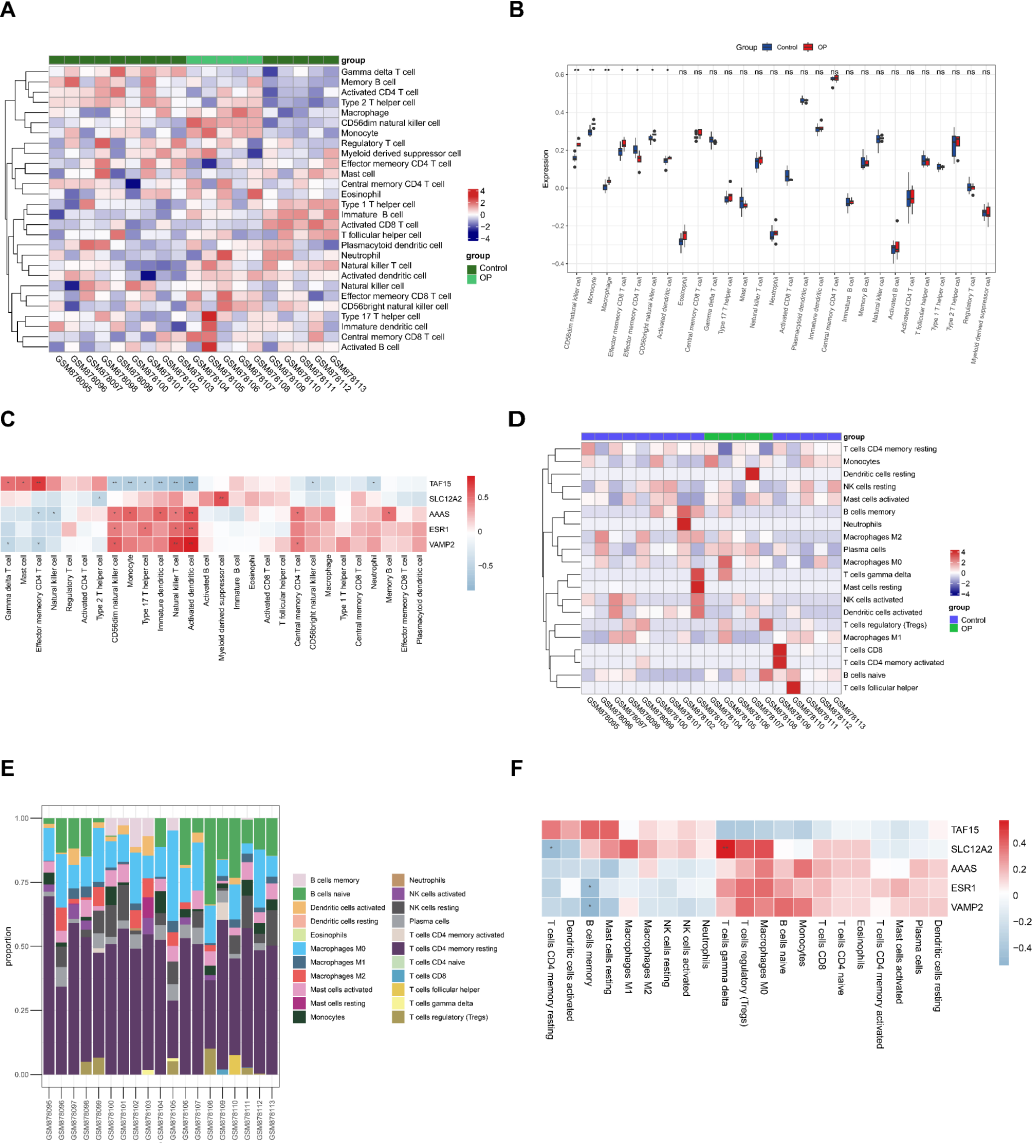
Immune infiltration analysis. (**A**) Heatmap of immune cell abundance in ssGSEA. The horizontal coordinate represents the samples and the vertical coordinate represents the immune cells. Red represents high infiltration and blue represents low infiltration. (**B**) Boxplot of 28 immune cells abundance. Blue represents control and red represents OP. (**C**) The correlation heat map between key genes and immune cells in ssGSEA. Red represents positive correlation and blue represents negative correlation. (**D**) Heatmap of immune cell abundance in Cibersort. (**E**) Bar graph of 22 immune cells percentages. Horizontal coordinates represent samples, vertical coordinates represent percentages, and colors represent immune cells. (**F**) The correlation heat map between key genes and immune cells in Cibersort

Studies have confirmed that immune disorders regulate bone metabolism [33]. Therefore, immune infiltration analysis was performed by ssGSEA and CIBERSORT algorithms. Heatmap showed different immune cell infiltration abundance based on ssGSEA (Fig. 6A). Among 28 immune cells, CD56dim natural killer cells, monocytes, macrophages, effector memory CD8 T cells, CD56bringht natural killer cells and activated dendritic cells had higher abundance in OP than control, while effector memory CD4 T cells trended in reverse direction (Fig. 6B). Correlation analysis between key genes and immune cells showed that *TAF15* expression was positively linked to effector memeory CD4 T cell and negatively linked to activated dendritic cell. *TAF 15* probably had multiple roles in immune infiltration regulation in OP. *VAMP2* was positively associated with natural killer T cell and activated dendritic cell. *SLC12A2* was negatively correlated with immature dendritic cell (Fig. 6C).

Heatmap showed different immune cell infiltration abundance based on the CIBERSORT (Fig. 6D). The bar chart of 22 immune cells showed that NK cells, B cells and T cells occupy a larger proportion (Fig. 6E). Correlation analysis between key genes and immune cells showed that *SLC12A2* was positively associated with T cell gamma delta and negatively associated with T cell CD4 memory resting. *SLC12A2* likely has multiple regulatory effects on T cells. In addition, both *ESR1* and *VAMP2* were negatively linked to B cell memory (Fig. 6F). *ESR1* and *VAMP2* might have a synergistic role in regulating B cell memory.

### The mRNAs-miRNAs and TFs-mRNAs network

**Fig. 7:**
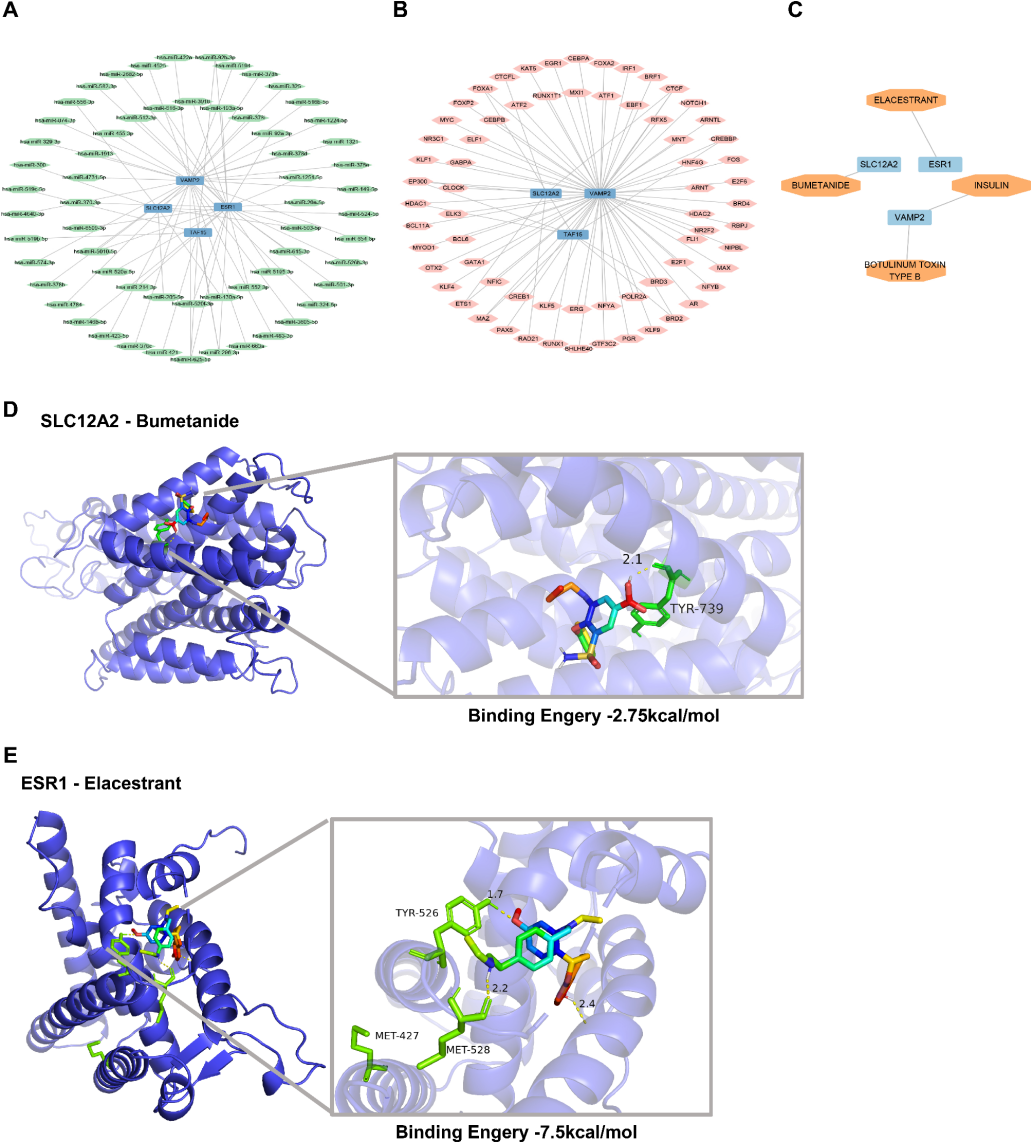
The mRNA-miRNAs, TFs-mRNA, mRNA-Drugs network and molecular docking. Blue represents key genes, green represents miRNAs, pink represents transcription factors, and orange represents small molecule drugs. (**A**) The mRNA-miRNAs network. (**B**) The TFs-mRNA network. (**C**) The mRNA-drugs network. (**D**, **E**) Molecular docking showed the binding relationship of *SLC12A2*-bumetanide (**D**) and *ESR1*-elacestrant (**E**)

The mRNAs-miRNAs and TFs-mRNAs network were built by cytoscape. The has-miR-625-5p and has-miR-296-3p could interact with *ESR1*, *VAMP2* and *TAF15* respectively (Fig. 7A and Table. S6). The CTCF and EP300 could interact with *SLC12A2*, *TAF15* and *VAMP2* respectively (Fig. 7B and Table. S7).

### The mRNA-drugs network and molecular docking

The small molecular drugs obtained through DGIdb database screening included elacestrant, bumetanide, botulinum toxin type b and insulin (Fig. 7C and Table. S8). Candidate drugs were further screened by affinity binding energy in molecular docking. Molecular docking displayed that bumeranide binds to sites in the active pocket of *SLC12A2* with a binding energy of -7.5 kcal/mol and elacestrant binds to sites in the active pocket of *ESR1* with a binding energy of -2.75 kcal/mol (Fig. 7D and E).

### Verification of the mRNA expression of key genes

**Fig. 8:**
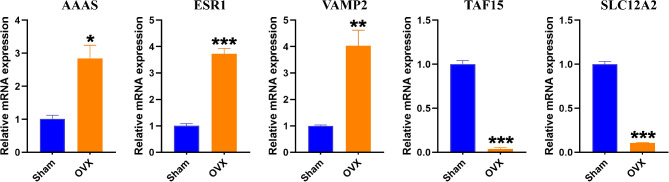
The qRT-PCR of mRNA expression of key genes. (**A**) *AAAS* (**B**) *ESR1* (**C**) *VAMP2* (**D**) *TAF15* (**E**) *SLC12A2*. The *P < 0.05, **P < 0.01, ***P < 0.001

The qRT-PCR revealed that mRNA expression of *AAAS*, *ESR1* and *VAMP2* were higher, and *SLC12A2* and *TAF15* were lower in OP than control group (Fig. 8).

## Discussion

OP is a metabolic bone disease that occurs in postmenopausal women and older men [3, 34]. The pathogenesis of OP is complex and unclear [35–37]. The clinical diagnosis of OP mainly relies on DXA to detect BMD, but DXA is insensitive to early bone loss and unable to assess the severity of bone loss [13]. OP is mainly treated with drugs, but all have limitations and adverse effects [14]. Recent studies have shown that ERS and MD participate in bone metabolism, especially OP [38–41]. There is numerous crosstalk between ERS and MD [42, 43]. However, little attention has been paid to ERS and MD in OP. In this study, bioinformatics analysis and machine learn were used to screen biomarkers related to ERS and MD, and molecular docking was performed to screen potential drugs in OP.

In this study, DEGs were screened from OP microarray data by bioinformatics analysis. The hBMSCs are difficult to obtain, and GSE35959 is the largest dataset on hBMSCs. By differential analysis of GSE35959, 122 ERS&MDRDEGs were obtained.

GO enrichment analysis showed that ERS&MDRDEGs participated in several vital biological processes such as osteogenesis, mineral deposition and lipid metabolism. Meanwhile, ERS&MDRDEGs were enriched in the estrogen signaling pathway, which is the major bone metabolism pathway in OP [44]. Enrichment analyses indicated that ERS&MDRDEGs were associated with many essential biological processes and play critical roles in OP.

The 5 key genes (*AAAS*, *ESR1*, *SLC12A2*, *TAF15* and *VAMP2*) were gained by WGCNA and machine learning. The OVX mice are commonly used as preclinical OP models. The differential expression of key genes in bone loss mice was verified again by qRT-PCR. The qRT-PCR results were basically consistent with chip data analysis. Meanwhile, ROC curves showed that key genes have superior diagnostic value for OP. Studies have found that *AAAS* mutations cause triple A syndromes including alacrima, achalasia, adrenal failure and progressive neurodegenerative disease, resulting in low BMD and OP [45]. It has been reported that oxidative stress can shorten the survival time and reduce osteogenic differentiation of BMSCs, thus aggravating bone loss caused by estrogen deficiency [46]. Meanwhile, oxidative stress can induce ERS and MD [47]. Mechanically, ESR1 activates the antioxidant pathway through Nrf2, promotes osteogenic differentiation and bone formation of BMSCs, and prevents valuable loss [46]. Current studies have shown that artesunate treated BMSCs-derived exosomes deliver SNHG7 via the *TAF15/RUNX2* axis to facilitate osteogenesis [48]. The mi-RNA185 can directly bind *VAMP2* to inhibit its expression, thereby inhibiting proliferation, invasion and metastasis of osteosarcoma cells [49]. The effect and mechanism of *SLC12A2* and *VAMP2* in OP have not been reported. In conclusion, key genes play critical roles in bone metabolism, and more experiments are needed to confirm their specific mechanism in OP.

Recent studies have shown that bone immune disorders are also involved in OP pathogenesis [33, 50]. In this study, immune infiltration analysis revealed a higher abundance of monocyte and macrophage in OP compared to control group. In OP, the differentiation of monocytes into osteoclasts increases, promoting bone resorption and leading to bone loss [51]. The macrophages polarize M1-like and transport oxidation-damaged mitochondria to BMSCs, resulting in abnormal metabolism, reduced osteogenic differentiation, and imbalance of bone homeostasis in OP [8]. Macrophages play a regulatory role in bone metabolism.

The mRNA-miRNA network showed that has-miR-625-5p and has-miR-296-3p can interact with *ESR1*, *VAMP2* and *TAF15* separately. Studies have found that quercetin promote proliferation and osteogenic differentiation of BMSCs by activating Wnt/β-catenin pathway through the H19/miR-625-5p axis [52], and miR-296 encourages osteoblast differentiation by up-regulating Cbfal [53]. The TFs-mRNA network showed that CTCF and EP300 can interact with *SLC12A2*, *TAF15* and *VAMP2* separately. However, roles of CTCF and EP300 about OP have not been reported, so their mechanism in OP need further exploration.

Molecular docking displayed that bumeranide-*SLC12A2* and elacestrant-*ESR1* with stable binding. Bumetanide, an organic compound with the chemical formula C17H20N2O5S, is mainly used as diuretic [54]. Studies have shown that bumetanide affects bone transformation and plasma parathyroid hormone levels [55]. Elacestrant was first oral estrogen receptor antagonist, approved by the FDA for late-stage breast cancer in 2023 [56]. The effects and mechanisms of bumeranide and elacestrant on ERS and MD in OP need to be further explored.

Despite some progress we have made in identifying key genes and potential drugs related to OP, there are still some limitations. First, by removing batch effects from the dataset, the interference of potential batch effects can be reduced, but it may result in some information loss. We need to be careful in interpreting the dataset bias and avoid exaggerating the results’ generalization. Meanwhile, due to the small number of samples in training set, overfitting may occur, which affects the generalization ability of external validation. Although we performed cross-validation to enhance robustness, the effects of potential biases and external variables are still difficult to exclude. More diverse and larger sample size datasets and more advanced algorithms are needed in the future to further validate the stability and adaptability of key genes. Meanwhile, more basic experiments and clinical studies are needed to validate the biological effects of key genes and potential drugs.

## Conclusion

In this study, 5 key genes related to ERS and MD in OP (*AAAS*, *ESR1*, *SLC12A2*, *TAF15* and *VAMP2*) were identified. The 2 small molecule compounds (bumetanide and elacestrant) might be potential drugs for ERS and MD in OP. This study provides strong support for understanding the pathophysiology of OP and developing new treatment strategies.

## Electronic supplementary material

Below is the link to the electronic supplementary material.

Supplementary Material 1

Supplementary Material 2

Supplementary Material 3

Supplementary Material 4

Supplementary Material 5

Supplementary Material 6

Supplementary Material 7

Supplementary Material 8

Supplementary Material 9

Supplementary Material 10

Supplementary Material 11

## Data Availability

The datasets analyzed during the current study are available in public databases such as GEO (https://www.ncbi.nlm.nih.gov/geo/), MSigDB database (https://www.gsea-msigdb.org/gsea/msigdb) and GeneCards (https://www.genecards.org/).
