# Supplementary material for: Identification of endoplasmic reticulum stress and mitochondrial dysfunction related biomarkers in osteoporosis
Source: Hereditas. 2025 Feb 14;162:21. doi: 10.1186/s41065-025-00387-7 (PMC11827247; doi:10.1186/s41065-025-00387-7)
Supplement: Supplementary file 4 — Supplementary Material 4 [file 41065_2025_387_MOESM4_ESM.docx]

**Table S1 GEO Microarray Chip Information**

|  | GSE35959 | GSE7158 | GSE56814 | GSE56815 |
| --- | --- | --- | --- | --- |
| Platform | GPL570 | GPL570 | GPL5175 | GPL96 |
| Type | Array | Array | Array | Array |
| Species | Homo sapiens | Homo sapiens | Homo sapiens | Homo sapiens |
| Tissue | Bone Marrow | Blood | Blood | Blood |
| Samples in OP group | 5 | 12 | 31 | 40 |
| Samples in Control group | 14 | 14 | 42 | 40 |
| Reference | PMID: 23028809 | PMID: 19223260 | PMID: 30056508 | PMID: 29330445 |

GEO，Gene Expression Omnibus；OP，Osteoporosis
